# Heterogeneity and associated factors of patients with polycystic ovary syndrome health behaviors: a latent class analysis

**DOI:** 10.1186/s12902-023-01385-4

**Published:** 2023-06-25

**Authors:** Ying liu, Yunmei Guo, Rui Ding, Xin Yan, Huiwen Tan, Xueting Wang, Yousha Wang, LianHong Wang

**Affiliations:** 1 Nursing Department, Affiliated Hospital of Zunyi Medical University, 563000 ZunYi, Guizhou China; 2 Nursing College, ZunYi Medical University, 563000 ZunYi, Guizhou China

**Keywords:** Health behaviors, Polycystic ovary syndrome, Latent class analysis

## Abstract

**Objective:**

Using latent class to analyze whether there are subtypes of health behaviors in patients with PCOS can be addressed using targeted interventions.

**Methods:**

October 2021 to June 2022, 471 PCOS patients were surveyed using the Health Promoting Lifestyle Profile Questionnaire. Latent class analysis (LCA) was used to identify subgroups of PCOS patients. Subsequent multinomial latent variable regressions identified factors that were associated with health behaviors.

**Results:**

A three-class subtypes was the optimum grouping classification: (1)High healthy behavior risk; (2)high healthy responsibility and physical activity risk; (3)low healthy behavior risk. The multinomial logistic regression analysis revealed that (1)Single (OR = 2.061,95% CI = 1.207–3.659), Education level is primary school or below (OR = 4.997,95%CI = 1.732–14.416), participants is student (OR = 0.362,95%=0.138–0.948), participants with pregnancy needs (OR = 1.869,95%=1.009–3.463) were significantly more likely to be in the high healthy behavior risk subtypes; (2)The older the age (OR = 0.953,95%=0.867–1.047) and the larger the WC (OR = 0.954,95%=0.916–0.993), participants is married (OR = 1.126,95%=0.725–1.961), participants is employed ( OR = 1.418,95%=0.667–3.012) were significantly more likely to be in the high health responsibility and physical activity risk subtypes.

**Conclusion:**

Patients with PCOS are a heterogeneous population with potential subtypes that may be suitable for customized multi-level care and targeted interventions.

## Introduction

Polycystic ovary syndrome(PCOS) is one of the most common endocrinopathy affecting reproductive aged women [1], with the prevelence of between 5–25% [2]. It is characterized by persistent anovulation and / or rare ovulation, folliculosis and hyperandrogenism. PCOS usually causes patients to experience menstrual disorders, infertility, obesity and other clinical symptoms [3], and even brings about an annual economic burden of about 8 billion US dollars [4]. Lifestyle management as an internationally recognized first-line treatment [5–7], it can effectively improve the clinical symptoms, physiological metabolism and psychological characteristics of PCOS patients. However, the effect of lifestyle management is greatly affected by the patient’s health behavior. That is, healthy lifestyle management depends on sustained active health behaviours [6, 8, 9]. And as a chronic disease, PCOS across the lifespan in patients and represents a major health and economic burden [10, 11]. Once there are disordered physiological changes, there will be a vicious circle [12]. As a result, an early and precise management is important for PCOS patients.

At present, numerous studies have confirmed that after lifestyle management, PCOS patients can reduce their average body weight about 10.6%, the incidence of menstruation can be reduced by 32.9%, and the average waist circumference can be reduced by about 3.41 cm [13, 14]. In addition, studies have also confirmed that the pregnancy rate of PCOS patients is significantly higher after lifestyle management, and depression, anxiety and stress are also improved [6, 15]. Although there have been researches on PCOS lifestyle management, the research to date has tended to focus on the overall impact of different diets, exercise types and/or compliance on PCOS patients rather than health behavior subtypes [15, 16]. Due to the individuals differences and heterogeneity, the rationality and scientificity of using the same intervention method remains to be studied, as they may require different lifestyle management and have different consequences. Therefore, it is necessary to further explore whether there are individuals or groups with different PCOS health behavior subtypes.

Latent class analysis (LCA) is a statistical way to uncover hidden clusters in data. LCA provides a framework for explaining population heterogeneity by identifying unobserved (latent) population subgroups that are inferred from observed variables, rather than describe the variability of a single variable [17]. Due to these advantages, it has been used in many fields in recent years, such as medicine, sociology, psychology, etc. [18–20], to identify unobserved subgroups, especially in identifying potential behavior patterns [21–23]. In medicine, LCA has been widely used to identify various disease phenotypes, disease heterogeneity and healthcare behaviours to identify the potential characteristics of various groups and provide evidence for clinical research [24]. In sociology, LCA are usually used to identify patterns of health risk behaviors, quality of life and family relationships in different populations, and provide guidance for the harmonious development of a healthy society [25–27]. In psychology, LCA is widely used to identify the potential subtypes of psychological distress and different subtypes of negative emotional symptoms, so as to understand the etiology and mechanism of the clinical and psychosocial characteristics of each potential subtype, which can provide information for more accurate and targeted intervention measures to meet the different needs of each subgroup [28, 29]. In this study, LCA will be used to cluster PCOS patients’health behaviors. It is a person-centered approach to classify smaller homogeneous clusters based on the probabilities of health behaviors reported by PCOS patients. To our knowledge, there have been no LCA studies on health behaviors in patients with PCOS.

To circumnavigate the“one size fits all” management strategies, we thus decided to use LCA to determine whether there are different subgroups of PCOS patients with different health behaviors. It is important to know the probability of a patient belonging to a certain group, and what characteristics differentiate them from other groups so that health promotion efforts can be targeted to change problematic health behaviors. The importance and originality of this study is to explores whether there are subtypes of health behaviors in patients with PCOS that can inform health promotion efforts, and if potential subtypes exist, it will help design targeted and effective prevention and intervention strategies to promote the long-term health of patients with PCOS.

## Materials and methods

### Participants and procedure

This study was a cross-section observational that recruited PCOS patients from the gynecology clinic department of affiliated hospital of Zunyi Medical University. This study was carried out from October 2021 to June 2022. The inclusion criteria include:①Patients aged ≥ 18 years; ②Meet the Rotterdam diagnostic criteria ③Be able to make self-report; ④Subjects informed consent. Exclusion criteria: ①Patients with psychoorganic diseases cannot complete self-report independently; ②Cognitive or other impairment, such as visual impairment, unable to complete self-report independently; ③Unwilling to participate in this study. Participation in the study was voluntary informed consent was provided that assured patients of the confidentiality of their collected data and the patients were allowed to decide to participate or not. Questionnaires were completed after diagnosis and treatment, with BMI and waist circumference measured by researchers and the rest self-reported by patients to assess their personal characteristics and health behaviors.

### Health behaviors

Two instruments were used to collect survey data. One of them was.

a self-reported questionnaire with two sections. The first section about sociodemographic datas. Each respondent independently completed that included age, nationality, residence, marital status, education, etc. And the second section on the demographically measurable indicators of patients including waist circumference, BMI, demand for pregnancy and so forth.

The second instrument was using the Chinese version of the Health Promoting Lifestyle Profile (HPLP-II) to measure health behaviors, first developed by Walker et al [30]. It measures health behaviors by focusing on self-initiated actions and perceptions that serve to maintain or enhance the level of wellness, self-actualization, and fulfillment of the individual. The HPLP-II is an instrument with a 52-item includes six health behavior subscales:(1) health responsibility (HR, 9 items), (2) nutrition (N, 9 items), (3)stress management (SM, 8 items), (4)physical activity (PA, 8 items), (5) interpersonal relationships (IR, 9 items), and (6)spiritual growth (SG, 9 items). All questions are scored on a 4-point Likert scale: Never (1 point), sometimes (2 points), often (3 points) and always (4 points). A mean of ≥ 2.50 was considered to be a positive response. A survey improved that the Cronbach’s alpha of 0.939 for Chinese, regarded as a reliability measure [31]. It has been validated in a number of randomized controlled trials and multi-center cross-sectional studies, and is considered to be a validity tool to assess the health behavior of Chinese people [31, 32].

### Statistical analysis

The statistical analyses were performed with SPSS 18.0 and Mplus, version 7.4. Firstly, SPSS was used for the data preprocessing. LCA was performed identify healthy behaviors subgroups (classes) of PCOS patients. To select the optimal number of classes, an exploratory approach was used, Which started with the most parsimonious 1-class model and successively increased the number of classes by one, until no improvement was observed. The best model was selected considered the following criteria:(1)Akaike Information Criterion (AIC, the lowest absolute values indicate good model fitness) (2)Bayesian information criterion (BIC,a lower BIC indicates better goodness of fit.) (3)Bootstrapped likelihood ratio test (BLRT) (use the k-1 and k class models test the null hypothesis to determine whether there is a statistically significant difference, k equaling the number of classes. Thus, the k class model is a better fitting model than k-1 class when the *P* < 0.05, whereas the P > 0.05 indicated that k-1 class is superior reflect the data) [33] (4)entropy(with higher values indicating a better fit, ranging from 0 to 1). PCOS patients were classified into their most likely latent class on the basis of HPLP-II. Subsequent multinomial latent variable regressions identified factors that were associated with health behaviors.

## Results

### Demographics characteristics

Based on the nadir criteria and the exclusion of unqualified questionnaires, 471 questionnaires were finally included. The characteristics of patients in the study are shown in Table 1. The mean age of the participants was 25.56 ± 4.54. BMI was 25.25 ± 4.79, WC was 82.67 ± 12.66. The subjects in the study include 399(84.7%) Han-nationality and 72(15.3%) Ethnic minority.

**Table 1:** Characteristics of the study sample.(N = 471)

Variable	N(%)
**Age(x ± s)**	25.56 ± 4.54
**BMI(x ± s)**	25.25 ± 4.79
**WC(x ± s)**	82.67 ± 12.66
**Nationality**	
Han-nationality	399(84.7%)
Ethnic minority	72(15.3%)
**Registered residence**	
Urban	247(52.4%)
Rural	224(47.6%)
**Marital status**	
Single	231(48.8%)
Married	230(49.0%)
Widowed/divorced	10(2.1%)
**Education**	
Primary School or Below	2(0.4%)
Junior and senior high school	254(53.9%)
College or above	215(45.6%)
**Occupation**	
Employed	130(27.6%)
Unemployed	57(12.1%)
Student	90(19.1%)
Others	194(41.2%)
**Per capita monthly household income(RMB)**	
< 5000	270(57.3%)
5000–10,000	152(32.3%)
≥ 10,000	94(10.4%)
**Pregnancy needs**	
yes	243(51.6%)
no	228(48.4%)

### Latent class analysis of HPLP-II

According to the latent class analysis, we identified 3 class of HPLP-II. The 3-class model showed the lowest AIC and adjusted BIC values, a statistically significant BLRT p-value, and large entropy. Table 2 further demonstrates the distribution of HPLP-II and conditional probabilities in each class.

**Table 2:** Model fit information for competing latent class models (N = 471)

Model	*k*	AIC	BIC	aBIC	Entropy	BLRT
2 C	13	2963.948	3017.961	2976.701	0.678	< 0.0001
3 C	20	**2921.068**	**3004.165**	**2940.689**	**0.731**	< 0.0001
4 C	27	2921.698	3033.879	2948.186	0.650	0.2381

Note: k = Number of Free Parameters; AIC = Akaike information criteria; BIC = Bayesian information criteria; aBIC = Adjusted Bayesian information criteria; BLRT = bootstrapped likelihood ratio test

**Fig. 1:**
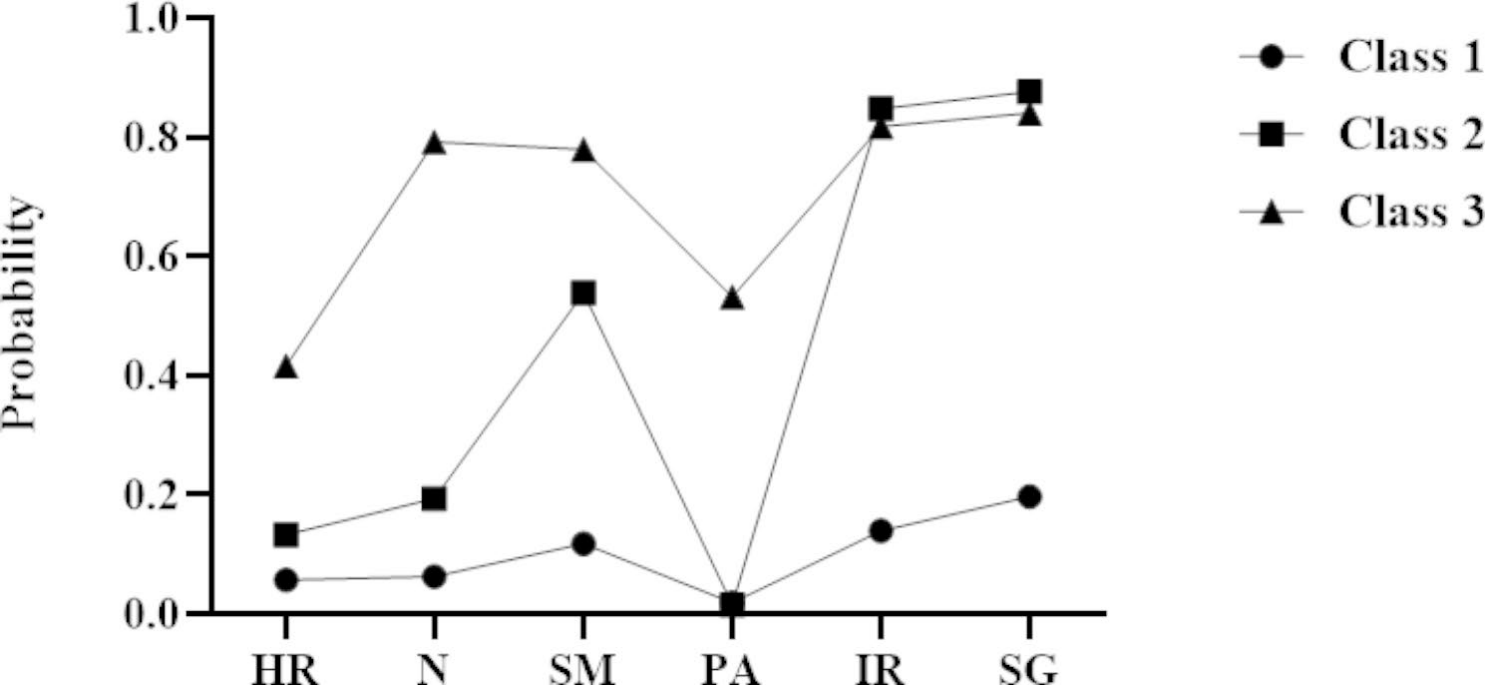
Health behavior characteristics of each class among PCOS(Note:Class 1 labeled “high healthy behavior risk”, Class 2 labeled “High health responsibility and physical activity risk”, Class 3 labeled “low healthy behavior risk”)

A graphical representation of the 3 classes among PCOS is shown in Fig. 1. The x-axis shows the health behaviors, and the y-axis represents the probability of having specific health behaviors within each class.

Class 1(labeled “high healthy behavior risk”, 39.3%) was characterized by PCOS with the high-risk healthy behavior, which was also the larger subgroup. They had the lowest scores in all six dimensions of health behavior.

Class 2(labeled “High health responsibility and physical activity risk”, 43.7% ) was characterized by PCOS with the highest physical activity risk and relatively high risk of health responsibility.

Class 3(labeled “low healthy behavior risk”,16.9% ) was characterized by PCOS with the low-risk healthy behavior. It consisted of highest health responsibility, nutrition, stress management and physical activity, relatively high of interpersonal relationships and spiritual growth.

### Multinomial logistic regression results for latent class analysis

To examine the effect of demographics on different classes, multinomial logistic regression analyses were performed, with “low healthy behavior risk” specified as the reference. Table 3 reveals that (1)Single(OR = 2.061,95% CI = 1.207–3.659), Education level is primary school or below (OR = 4.997,95%CI = 1.732–14.416), participants is student (OR = 0.362,95%=0.138–0.948), participants with pregnancy needs (OR = 1.869,95%=1.009–3.463) were significantly more likely to be in the high healthy behavior risk group; (2)The older the age (OR = 0.953,95%=0.867–1.047) and the larger the WC (OR = 0.954,95%=0.916–0.993), participants is married (OR = 1.126,95%=0.725–1.961), participants is employed (OR = 1.418,95%=0.667–3.012) were significantly more likely to be in the high health responsibility and physical activity risk group. BMI, registered residence and household income were not found to be associated with the latent classes of health risk behaviors.

**Table 3:** Multinomial logistic regression results of latent class(N = 471)

CLASS		high healthy behavior risk		High health responsibility and physical activity risk	
		OR	95%CI	OR	95%CI
**Age**		1.008	0.918–1.107	**0.953**	0.867–1.047
**WC**		0.985	0.918–1.07	**0.954**	0.916–0.993
**Marital status**	**Single**	**2.061**	1.207–3.659	1.405	1.083–1.917
	**Married**	4.388	2.158–7.837	**1.126**	0.725–1.961
	**Widowed/divorced**	-	-	-	-
**Education level**	**Primary School or Below**	**4.997**	1.732–14.416	1.694	0.581–4.942
	**Junior and senior high school**	1.232	0.636–2.385	0.567	0.298–1.077
	**College or above**	-	-	-	-
**Occupation**	**Employed**	1.006	0.463–2.186	**1.418**	0.667–3.012
	**Unemployed**	1.053	0.407–2.725	0.692	0.256–1.872
	**Student**	**0.362**	0.138–0.948	0.546	0.221–1.347
	**Others**	-	-	-	-
**Pregnancy needs**	**yes**	**1.869**	1.009–3.463	1.579	0.864–2.887
	**no**	-	-	-	-

Note:Table 3 presents the results of LCA multinomial regression analyses examining predictors of latent class membership with “low healthy behavior risk” group specified as the reference

## Discussion

The aim of this study was to use latent categories to analyze whether there are subtypes of health behaviors in patients with PCOS can be addressed using targeted interventions. Using LCA, 3 latent classes were identified: (1)high healthy behavior risk (39.3%) (2)High health responsibility and physical activity risk (47.3%) (3)low healthy behavior risk (16.9%). The majority of patients were classified into high health responsibility and physical activity risk (47.3%), with the lowest percentage of low health behavior risk categories (16.9%). All latent categories indicated that patients with PCOS were relatively better in the interpersonal relationships and spiritual growth dimensions. This is inconsistent with Anna L’s study and may be related to the adoption of different assessment instruments [34]. The lowest score in the physical activity dimension is consistent with the latest systematic review [35]. Overall, these findings suggest the emergence of meaningful subtypes of PCOS patients in the areas of health responsibility, nutrition, stress management, physical activity, interpersonal relationships, and spiritual growth.

The results of this study showed that the overall health behaviors of PCOS patients were poor. Among them, patients in Class1 were the most in need of improving health behaviors because they were at a low level in all six dimensions and they accounted for a larger proportion (39.3%). Consistent with other studies that concluded that health behaviors such as physical activity and diet were poorer in patients with PCOS [36, 37]. The results of this study further confirm the need for enhanced health behavior in Class1. Compared with the low health behavior risk class, those who were single, had primary school or below education, were students, and had a need to become pregnant were more likely to fall into Class1. A possible explanation for this is that according to social support theory and family system, married individuals are more likely to improve their health behaviors by communicating with their spouse [38]. In contrast, single/divorced/widowed individuals are more likely to feel lonely and pessimistic, which may contribute to their poorer health behaviors. Patients with low education level have limited knowledge of health hazards and risk perception, which affects their health behaviors [39]. The reasons why students are more likely to fall into Class1 may be related to the gradual increase in independent living and the multiple stressors of academic life creating an environment that supports engagement in risky health behaviors [40]. Surprisingly, the results of this study suggest that patients with a need for pregnancy are associated with high health behavior risk, which is inconsistent with the Stephenson’s study [41]. A possible explanation for this is that women with PCOS who have a need to conceive have concerns about fertility and have difficulty obtaining timely and reliable information to achieve their reproductive goals, resulting in poorer health behaviors [42].

At present, studies have demonstrated the link between a healthy lifestyle and metabolic health, and although the pathogenesis of metabolic or endocrine disorders involves several variants of different genes, all their components are influenced by lifestyle management [43–45]. In addition, studies have shown that health behaviors are negatively correlated with mental health problems [46, 47]. Therefore, PCOS as a complex disease in which patients maintain low health behaviors for a long time, may increase the incidence of metabolic diseases, cardiovascular diseases and mental health problems due to the interaction of hormonal, psychological and metabolic influences, but further confirmation is needed. For Class1 patients, when the patient is diagnosed with PCOS, the medical staff should progressively provide comprehensive health guidance to prevent risk by making lifestyle changes before they appear factors by making lifestyle changes before they appear. To encourage healthy behaviors, firstly, it is important to identify the underlying causes that hinder healthy behaviors in patients with PCOS. Then, explore the motivating factors, what resources they need, and how to provide these resources and help. Finally, patients should be followed up regularly to monitor various health behaviors to improve PCOS.

Class2 was the worst in terms of health responsibility and physical activity. Health responsibility is important for patients with PCOS because personal health behavior determines exposure to high-risk factors, while health responsibility determines personal health behavior. The present study confirmed a synergistic effect of health behaviors in PCOS patients, with lower health responsibility being associated with lower physical activity. In the current study it was shown that older, thicker waist circumference, married and employed patients were more likely to fall into Class2. There are several possible explanations for this result. First, older, married and employed patients may have a more stressful life and work, leading them to distract from their health responsibility and lack of physical activity time [48]. Second, studies have demonstrated a positive correlation between physical activity and waist circumference, so patients with thicker waist circumference may also be less physically active [8]. Therefore, medical staff should focus on health responsibilities and physical activity in the older, married and employed population with PCOS. With evidence suggesting that low levels of physical activity increase the risk of major chronic diseases and all-cause mortality in the general population, maintaining exercise training not only reduces the risk of cardiovascular disease, but also provides direct endogenous cardiovascular protection [49–51]. Therefore, Class2 may have a higher incidence of cardiovascular disease compared to the other two class. In summary, for Class2 patients, medical staff can enhance physical activity by promoting PCOS-related health education tools, by increasing the sense of responsibility for health maintenance and motivation to cope with the disease in patients with PCOS.

The Class3 is relatively better in all dimensions of health behavior and patients actively participate in various health behaviors. However, Class3 only accounted for 16.9% of the patients in this study, indicating that the health behavior of PCOS patients still needs to be improved. The study by Sedighi et al. also showed that the healthy behaviors of PCOS patients is worse than that of general healthy women [52]. Class3 patients may reduce the clinical symptoms, improve endocrine metabolism and mental health in patients with PCOS, and this effect is attributed to the protective and therapeutic effects of healthy behaviors in patients with PCOS [53, 54]. For Class3, it is most important to encourage patients to maintain their current healthy behaviours over the long term. Therefore, medical staff can emphasize to patients the importance of maintaining long-term healthy behaviors for PCOS, so that PCOS patients can fully understand healthy behaviors through information, and can be more motivated and confident to formulate focused behaviors to obtain greater health benefits.

In conclusion, this study found that patients with PCOS are a heterogeneous population consisting of unique subtypes that may lend themselves to customized multilevel care. This finding will help to develop targeted intervention programs for patients with PCOS to improve their health status. In addition, patients with PCOS are poorer in health behaviors overall, especially in terms of health responsibility and physical activity. Unexpectedly, BMI, ethnicity, residence, and household income were not found to be associated with subtypes of PCOS patients, and more research is needed to clarify this relationship.

## Strengths and limitations

To our knowledge, this is the first study using latent class to analyze health behaviors in patients with PCOS in order to provide targeted intervention strategies. Secondly, the previous studies on the health behavior of PCOS mostly focus on diet and exercise. The HPLP-II questionnaire used in this study contains six dimensions, and the survey is relatively comprehensive. Despite the interesting implications of these findings, it is important to highlight some limitations. A key limitation of this study is that the sample represents only one hospital, rather than the population of PCOS patients in China. Thus, future studies including PCOS patients from other provinces across China and other countries are needed to verify the results generated in this study. Furthermore, The health behaviors of all participants were self-reported and thus there may be reporting bias.

## Data Availability

The datasets used and/or analysed during the current study available from the corresponding author on reasonable request.
